# Comparing Web-Based and In-Person Educational Workshops for Canadian Occupational Therapists and Understanding Their Learning Experiences: Mixed Methods Study

**DOI:** 10.2196/31634

**Published:** 2022-01-04

**Authors:** Sungha Kim, Ilana Bayer, Rebecca Gewurtz, Nadine Larivière, Lori Letts

**Affiliations:** 1 School of Rehabilitation Science McMaster University Hamilton, ON Canada; 2 Department of Pathology and Molecular Medicine McMaster University Hamilton, ON Canada; 3 Department of Medicine University of Sherbrooke Sherbrooke, QC Canada

**Keywords:** online education, occupational therapy, occupational therapist, continuing education

## Abstract

**Background:**

The Do-Live-Well (DLW) framework is an occupation-focused health promotion approach. Occupational therapists (OTs) have been interested in training opportunities regarding this framework. Traditionally, in-person continuing educational interventions are the main way that OTs obtain knowledge, but web-based learning has become popular among health care professionals. However, its effectiveness and learners’ experience in web-based learning have not been well-studied in occupational therapy education.

**Objective:**

This study aims to evaluate the effectiveness of the web-based and in-person educational DLW workshops for Canadian OTs and to understand their experiences in both workshop types.

**Methods:**

An explanatory sequential mixed methods study design was used, where quantitative data were collected first, then qualitative data were used to explain the quantitative findings. A quasi-experimental design and interpretative description methodology were used in the quantitative and qualitative phases, respectively.

**Results:**

Quantitative results were as follows: a total of 43 OTs completed pre-, post-, and follow-up evaluations (in-person group: 21/43, 49%; web-based group: 22/43, 51%). Practice settings of the participants varied, including geriatric, hospital, long-term, mental health, pediatric, and primary settings. The primary outcome was as follows: there were no statistically significant differences in knowledge changes at the 3 time points (*P*=.57 to *P*=.99) between the groups. In the web-based group, the knowledge scores at follow-up were lower compared with the posttest results, meaning that knowledge gain was reduced over time (*P*=.001). The secondary outcomes were as follows: there were statistically significant differences between the groups in factors influencing DLW adoption at posttest (*P*=.001) and in satisfaction with the workshop (*P*<.001) at posttest in favor of the in-person group. Qualitative results were as follows: a total of 18 OTs (9/18, 50% from each group) participated in an individual interview. Five themes were identified regarding learners’ workshop experiences: relevance to their practices and interests may improve learning, a familiar learning environment may facilitate learning, synchronous in-person interaction is valuable in the learning process, ease of access to learning should be considered, and flexibility in web-based learning can be both beneficial and challenging.

**Conclusions:**

The quantitative results of this study reported no difference in knowledge acquisition between the in-person and web-based groups, indicating that web-based education is as effective as in-person workshops. However, participants’ satisfaction with the workshop was statistically significantly higher for the in-person workshop. The qualitative findings described the participants’ perceived benefits and challenges of each educational format. The participants in both the web-based and in-person workshop groups valued in-person interactions in learning, but the participants in the web-based workshop group expressed web-based learning lacked in-person-like interactions. Thus, adding synchronous in-person interactions to web-based learning may improve learners’ educational experiences in web-based occupational therapy and continuing education.

## Introduction

### Background

Each day, human beings engage in various occupations, defined as sets of activities for purposes, such as self-care, leisure, and productivity that are a core concept of occupational therapy [1]. Occupation-focused frameworks are used by occupational therapists (OTs) to understand occupational issues, enabling the provision of services that are responsive to the needs and goals of the clients [2]. The Do-Live-Well (DLW) framework is an evidence-based Canadian health promotion approach developed by OTs [3]. The key message of the DLW framework is that engaging in daily patterns of activity that allow for an optimal range of experiences with sufficient personal and social support can lead to a wide range of positive health and well-being outcomes [3]. Despite interest in this relatively new framework from OTs around the world, continuing education to support the adoption of the framework in practice has been limited to only certain areas of Canada, including Quebec and Ontario. On the basis of requests nationally and internationally, the developers of the framework identified a need to provide educational opportunities to meet these expanding learning needs.

The importance of health care professionals engaging in continuing education activities to advance their professional knowledge and expertise has long been emphasized [4]. OTs have used continuing education as a primary resource to maintain and improve their knowledge, ensure clinical competency, and pursue personal development [5,6]. The importance of continuing education in occupational therapy practice has been addressed in literature [7-9]. Although the most common type of continuing education for OTs is through in-person delivery methods such as conferences, presentations, and seminars or workshops [6], web-based education has become increasingly popular in health care professions across the world [4].

In this study, the term *web-based learning* was defined as “learning experiences via the use of some technology” [10]. Although cultural and technological adaptations are required to implement web-based learning [11,12], the advantages of this web-based delivery modality have been shown in health professional education, such as easy accessibility to learning without geographical restrictions, customized learning pace, and multimedia use [11-14]. In particular, the COVID-19 outbreak in December 2019, leading to public health restrictions through 2020 and 2021, has dramatically changed the means of delivering knowledge from traditional in-person learning to web-based methods [15]. This indicates that web-based learning is no longer simply an option but rather an essential educational delivery route. Although the importance and availability of web-based education in occupational therapy has been emerging since the beginning of the 21st century [16], the effectiveness of web-based education as a continuing educational opportunity compared with in-person education for OTs has not been well-studied. A systematic review comparing the effectiveness of web-based and traditional in-person learning reported little or no difference in the knowledge, behavioral changes, or skills of health professionals [17]. However, these results may not be definitively generalized to occupational therapy education because only a small proportion of study participants were OTs (only 8% to 11% of OTs in one randomized controlled trial) [17]. Furthermore, although the existing studies provide quantitative results in terms of the effectiveness of web-based and in-person learning, they lack an understanding of how the participants experienced these educational delivery methods. This understanding of what does or does not work well in both educational methods may help educators in occupational therapy improve future learning environments. Thus, research is needed to compare the effectiveness of web-based and in-person education delivery methods and to understand the learning experiences of the participants in continuing occupational therapy education.

### Objectives

The objective of this study is to compare the effectiveness of a web-based DLW workshop with an in-person model for Canadian OTs and to understand the learners’ experience of participating in both web-based and in-person workshops. The primary research questions of this study are as follows: *What is the effectiveness of the web-based DLW workshop compared with the in-person DLW workshop?* and *What are the perceived benefits and challenges of participating in both educational delivery methods?*

## Methods

### Study Design

#### Overview

**Figure 1:**
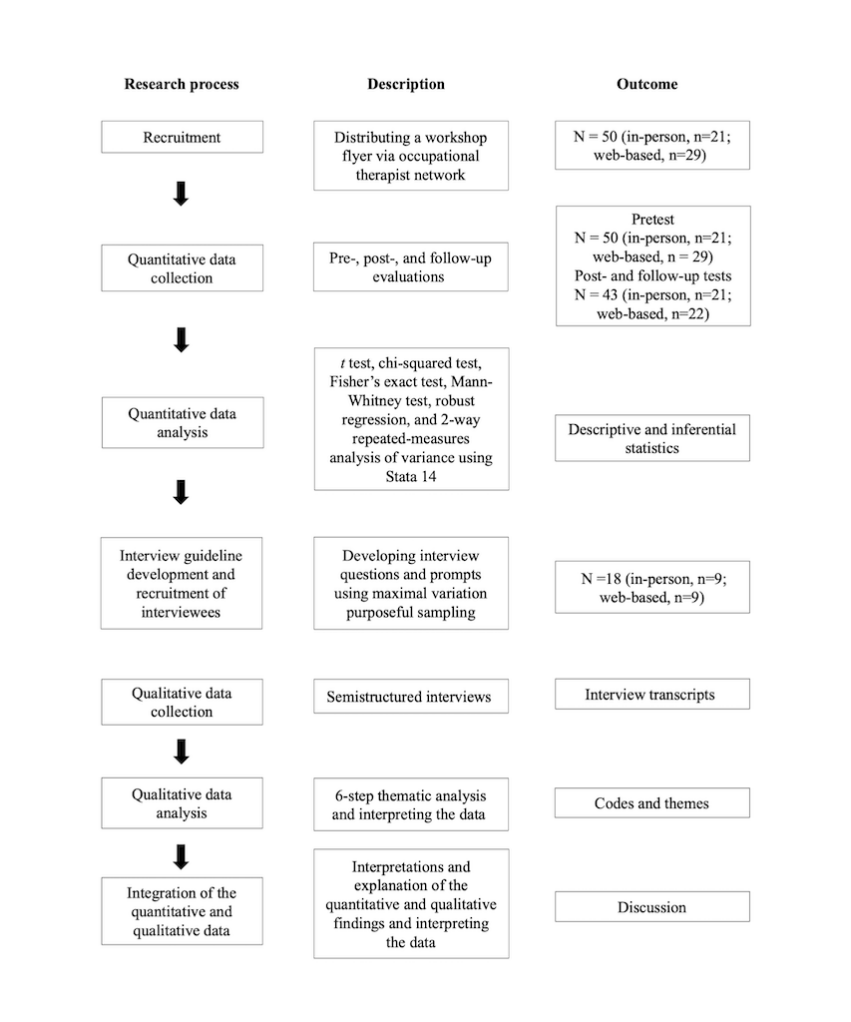
Overview of the study design, including the research process, description, and outcome for each stage.

This study was approved by the Hamilton Integrated Research Ethics Board (Project 4114). An explanatory sequential mixed methods study design was used to evaluate the effectiveness of web-based and in-person DLW workshops and to understand the experiences of the participants in learning about the framework [18]. This study consisted of 2 phases, in which quantitative data were collected first and then qualitative data were used to expand on the findings from the quantitative data. A visual diagram of the study process is presented in Figure 1.

#### Quantitative Phase

A pre-, post-, and follow-up quasi-experimental design was used to compare the immediate and subsequent outcomes of the web-based workshop with those of the in-person workshop. Participants were not randomly assigned because of geographical limitations.

#### Qualitative Phase

An interpretative description approach [19] was used to understand the learners’ perceived benefits and challenges of participating in the workshops. Interpretative description was considered appropriate for use because it allows for a flexible approach to capturing the experiences of the participants and for researchers to apply research findings to practice [19].

### Participants

#### Quantitative Phase

Participants were Canadian OTs who were offered to attend either the web-based or in-person DLW workshop, and they selected one of the learning formats to attend. We recruited participants by distributing a research flyer via Canadian OT communities and offered the workshop free of charge as part of the study participation. Canadian OTs practicing in any setting were eligible to participate in this study because the DLW framework is designed to be applied to people of any age, health condition, capacities, and occupational challenges. The total target sample size was 51; this estimate was based on an expected effect size of 0.9 gain in knowledge [20], where a power of 0.8, α of .05, and a 20% dropout rate were applied. A workshop flyer was posted on the Canadian Association of Occupational Therapists website, and the DLW team members shared the flyer with colleagues in their network to recruit eligible participants.

#### Qualitative Phase

Although there are no guidelines for calculating sample size in qualitative research [21], and interpretative description can be performed with almost any sample size [19], it is recommended to have at least 12 participants to reach data saturation in this type of design [22]. We recruited web-based and in-person workshop participants for a semistructured, 1:1 interview. We sent an invitation to all workshop participants via email to seek participation in an interview 3 months after the workshop. We hoped that we would gain various perspectives from participants in different clinical settings who used the DLW framework to varying degrees regardless of their education, work experience, and gender [23].

### Workshop Description

Both the web-based and in-person workshops consisted of 4 sessions (the schedule is shown in Textbox 1). Workshop content was scripted to ensure that both web-based and in-person workshops delivered the same content. The in-person workshop was a single-day, 8-hour workshop, and the web-based workshop was planned to last 4 weeks, also taking approximately 8 hours. A problem-based learning (PBL) approach was incorporated to facilitate a learner-centered learning environment for both formats. For example, participants in both workshops were divided into 5 groups according to the case scenario they chose, and they had a chance to answer reflective questions through discussions. Each group in the in-person workshop watched the video case scenario assigned to them in a separate space. To meet the purpose of this study, we limited the interactions provided in the web-based workshop to asynchronous components, recognizing that synchronous activities using technology are possible, but this was not the focus of our study. Although the web-based workshop was asynchronous and prerecorded, asynchronous discussion forums were provided on the web to give learners an opportunity to interact and share their perspectives with one another as well as with educators with expertise in the DLW framework. The DLW team members monitored both web-based and in-person group discussions and answered questions raised during the discussions. Although the learners in the in-person workshop could immediately hear the answers to their questions, the web-based learners could not receive immediate answers to their questions because of the nature of asynchronous web-based learning. Participants in the in-person group received a printed workbook, whereas the web-based group could download the same content electronically. The details of the workshop development process are described elsewhere [24].

**Workshop schedule.**

**Introducing instructors, participants, and learning and teaching approach**

- Session 1
  Introducing case scenarios
  Health promotion and health and well-being outcomes
- Session 2
  Introduction of the Do-Live-Well framework
  Dimensions of activity
- Session 3
  Activity patterns
  Social and personal support
- Session 4
  Application of the Do-Live-Well framework
  Large group case scenario discussions
- Wrapping up
  Question and answer and reflection
  Postevaluation

### Data Collection

#### Quantitative Phase

We developed the pre- (Multimedia Appendix 1), post- (Multimedia Appendix 2), and follow-up (Multimedia Appendix 3) questionnaires specifically for this study through a literature review and consultation with 4 occupational therapy research experts from the DLW research team. The purpose of the consultation was to ensure that the appropriate questions were included to measure the workshop outcomes. Three levels of the training evaluation model by Kirkpatrick and Kirkpatrick, including reaction, learning, and behavior, were used to decide on the content of the questionnaires [25]. The questionnaires at each time point consisted of slightly different content packages (Textbox 2) but aimed to capture a comprehensive understanding of the effectiveness of the workshop. We incorporated the key constructs of the diffusion of innovation model [26] into the questionnaire, particularly for questions about factors influencing DLW adoption. This was intended to ensure a comprehensive evaluation of the appropriate parameters to determine the potential for adopting the DLW framework among OTs. The diffusion of innovation model explains how new knowledge (innovation) is disseminated in a certain social system over time, and the main constructs used are attributes of innovation, communication channels, and the social system [26]. After developing the initial versions of the questionnaires, the researchers pretested them qualitatively with 4 graduate students in the rehabilitation science program at McMaster University. The questionnaires were refined based on the feedback from the students and discussions with the DLW research team members. For example, the level of knowledge questions was adjusted, and more detailed instructions were added.

**Questionnaire content.**

**Pretest**

- Part 1: background information about the participant
- Part 2: current status of the use of the Do-Live-Well (DLW) framework
- Part 3: factors influencing DLW adoption
- Part 4: knowledge questions

**Posttest**

- Part 1: factors influencing DLW adoption
- Part 2: knowledge questions
- Part 3: satisfaction with the workshop

**Follow-up test**

- Part 1: current status of the use of the DLW framework
- Part 2: factors influencing DLW adoption
- Part 3: knowledge questions

#### Primary Outcome

The primary outcome was knowledge of the DLW framework. The DLW research team tested how much the participants knew about the DLW framework at 3 time points (pre-, post-, and 3-month follow-up) through 2 multiple-choice questions and 8 true-or-false questions. Each question had a value of 1 point for a correct answer; if a respondent answered all questions correctly, they earned 10 points. The participants were asked to complete the preworkshop questionnaire 1 week before the workshop to evaluate their baseline level of knowledge of the DLW framework. The participants then were required to complete the postworkshop questionnaire immediately following the workshop, and 3 months after the workshop the participants were asked to complete the follow-up questionnaire.

#### Secondary Outcomes

The secondary outcomes included the following: (1) changes in factors influencing DLW adoption, (2) satisfaction with the workshops, and (3) current use of the DLW framework. For factors influencing DLW adoption, the questions asked were about the advantages, compatibility, complexity, trialability, and observability of DLW use [26]. The participants also evaluated their communication channels, social system, and intentions for DLW use. All participants were asked to complete their evaluations at 3 time points (pre, post, and 3-month follow-up). The questionnaire included 10 questions, a 6-level Likert scale (1=strongly disagree to 6=strongly agree), and the total score ranged from 10 to 60. The core ideas of the questionnaire were the same for the pre-, post-, and follow-up questionnaires, with the exception of 1 question regarding the participants’ desire to apply the DLW framework that was removed for the follow-up test. The participants were asked to score their satisfaction with their workshop experience immediately after the workshop. The satisfaction questionnaire consisted of 16 questions, with a Likert scale ranging from 1 (strongly disagree) to 7 (strongly agree), and its total score ranged from 16 to 112. The following are some of the example questions that were included: the accessibility of the workshop was convenient, the learning environment encouraged me to actively participate in learning, and the time frame of the workshop was appropriate. Finally, the participants were asked about their current use of the DLW framework by answering a *yes or no* question in both the pretest and follow-up questionnaires. They were also asked about the frequency with which they had used the DLW framework with their clients and at an organizational level, where 0 indicated *never use it* and 10 indicated *use it all the time*.

#### Qualitative Phase

The first author (SK) developed the qualitative interview guide based on the findings from the follow-up quantitative data analysis. The goal of this qualitative phase was to understand what worked well and what did not work well for participants in both learning formats by acquiring a comprehensive understanding of the participants’ learning experiences. The interview questions focused on exploring the experiences of each participant in the workshop, including facilitators and challenges of participating in the workshop and engaging with the workshop content, as well as recommendations for future workshops. Each interview lasted 40-60 minutes. Owing to the COVID-19 pandemic, all participants were interviewed on the web using the videoconferencing platform Zoom. The interviews were audio- and video-recorded with the consent of the participants.

### Data Analysis

#### Quantitative Phase

All statistical analyses were conducted using Stata version 14 (StataCorp) [27]. Descriptive statistics were generated to present the characteristics of the participants and the variables of interest. The 2-tailed *t* test was used to find the differences in the mean total scores of the normally distributed variables between the 2 groups. If the variable was not normally distributed, the Wilcoxon-Mann-Whitney test was conducted. To find differences in categorical variables between the 2 groups, the chi-square test was used, and the Fisher exact test was applied in the analysis of small samples. Robust regression was conducted as an alternative to the analysis of covariate and linear regression because of the violation of normality and homogeneity of variance assumptions, respectively. Any statistically significant differences over time in the variables was found using 2-way repeated-measures analysis of variance.

#### Qualitative Phase

The interviews were transcribed verbatim by the first author (SK), and data analyses were supported using NVivo 12 (QSR International) [28]. We followed the 6-step analytical process described by Braun and Clarke [29]. This process included the following: familiarizing with the data through repeated readings, developing codes, grouping codes into themes, reviewing themes, generating definitions and names of the themes, and writing a report [29]. The first author read all transcripts several times and immersed herself in the data. Then, she generated initial codes relevant to the primary goal of the qualitative phase, which was to understand the benefits and challenges of participating in a web-based or in-person workshop. When generating the themes, the researchers realized that participants in both groups had some experience with both formats, although not in the DLW workshop. For example, participants in the web-based group had prior experience with in-person learning and shared various perspectives on the benefits and challenges of participating in both formats. Thus, rather than generating themes comparing the experiences of participants in the web-based and in-person workshop, we generated themes describing the comprehensive perspectives and experiences of the participants regarding both formats. The first author then presented the data analysis process and reported the initial themes to the research team. The themes were refined and finalized through discussions among the research team.

To establish the credibility of the findings, the first author wrote reflective notes for each interview participant and discussed with the research team whether the identified themes answered the research questions [30]. Furthermore, detailed descriptions of the research methods were provided to ensure the dependability of the qualitative findings [30].

## Results

### Quantitative Data: Participant Characteristics

Initially, 50 OTs agreed to participate in the study (in-person group: 21/50, 42%; web-based group: 29/50, 58%). In total, 6 participants did not complete both the post- and follow-up evaluations. One participant did not complete the postevaluation, and another participant did not complete the follow-up evaluation. Because all evaluations were performed anonymously, it was impossible to personally contact those who did not complete the post- and follow-up evaluations to ask them why they did not complete the evaluations. Although we sent multiple emails to remind the participants of the evaluations, no one sent an email stating that they could not complete the evaluations. Thus, data comparing 21 in-person and 22 web-based workshop participants have been presented. There was no statistically significant difference in demographic characteristics between the 2 groups. The detailed characteristics of the participants are presented in Table 1.

**Table 1:** Participant characteristics.

Variables			In-person (n=21)		Web-based		Total		*P* value
Age (years), mean (SD)			39.29 (11.1)		38.3 (9.70)^a^		38.79 (10.32)^b^		.86
**Sex, n (%)**			21 (100)		22 (100)		43 (100)		.99
	Female	21 (100)		21 (95)		42 (98)			
	Male	0 (0)		1 (5)		1 (2)			
**Education level, n (%)**			21 (100)		29 (100)		50 (100)		.74
	BScOT^c^	4 (19)		7 (24)		11 (22)			
	MScOT^d^	17 (81)		22 (76)		39 (78)			
Overall years of experience as an occupational therapist, mean (SD)			13 (11.73)		12.46 (8.64)		12.69 (9.94)		.80
Years of practice in the current setting, mean (SD)			8.28 (9.89)		6.26 (6.33)		7.11 (7.99)		.64
**Resources used to learn about DLW^e^** **before the workshop, n (%)**			21 (100)		29 (100)		50 (100)		.05
	Journal	0 (0)		1 (3)		1 (2)			
	Lecture	1 (5)		2 (7)		3 (6)			
	Website	8 (38)		8 (28)		16 (32)			
	>1 of the above	6 (29)		2 (7)		8 (16)			
	None of the above	6 (29)		15 (52)		21 (42)			
**Practice setting, n (%)**			21 (100)		29 (100)		50 (100)		.46
	Geriatric	1 (5)		3 (10)		4 (8)			
	Hospital	1 (5)		3 (10)		4 (8)			
	Long-term	1 (5)		1 (3)		2 (4)			
	Mental	10 (48)		8 (28)		18 (36)			
	Pediatric	1 (5)		2 (7)		3 (6)			
	Primary	3 (14)		3 (10)		6 (12)			
	Private	1 (5)		0 (0)		1 (2)			
	None of the above	2 (10)		9 (31)		11 (22)			
**Preference, n (%)**			21 (100)		29 (100)		50 (100)		.65
	In-person	17 (80)		20 (69)		37 (74)			
	Web-based	2 (10)		6 (21)		8 (16)			
	None	2 (10)		3 (10)		5 (10)			
**Use of the DLW in practice, n (%)**			21 (100)		29 (100)		50 (100)		.17
	Yes	2 (10)		0 (0)		2 (4)			
	No	19 (90)		29 (100)		48 (96)			

^a^n=22.

^b^N=43.

^c^BScOT: Bachelor of Science in Occupational Therapy.

^d^MScOT: Master of Science in Occupational Therapy.

^e^DLW: Do-Live-Well.

### Quantitative Data: Primary Outcome

#### Effects of the Workshops on Knowledge Regarding the DLW Framework

At baseline, the in-person group (n=21) reported a mean of 5.48 (SD 1.75) out of 10 on their knowledge of the DLW framework, whereas the web-based group (n=29) reported a mean of 5.39 (SD 1.69) out of 10, meaning the participants knew approximately half of the core concepts of the DLW framework that were tested in the knowledge questionnaire. The *t* test showed no statistically significant difference between the groups at baseline (*P*=.87).

Immediately following the workshop, the participants who attended the in-person workshop reported a mean of 7.62 (SD 0.22) of 10, whereas the participants in the web-based workshop reported a mean of 7.81 (SD 0.27) of 10. There was no statistically significant difference in knowledge regarding the DLW framework between the 2 groups immediately following the workshop (*P*=.57).

Similarly, at the follow-up evaluation, there was no statistically significant difference in knowledge regarding the DLW framework between the groups (*P*=.99). The in-person group reported a mean of 7.05 (SD 1.12) of 10 and the web-based group had a mean of 6.77 (SD 1.80) of 10.

Regarding the knowledge differences over time between the web-based and in-person workshops, the Mauchly test of sphericity validated the use of the 2-way repeated-measures analysis of variance (*P*=.63). There was no statistically significant interaction between the type of workshop and time regarding knowledge of the DLW framework (*F*_2,48_=0.90; *P*=.41). The main effect for the workshop type was not statistically significant (*F*_1,48_=0.15; *P*=.70), meaning that there was no difference in knowledge means between the in-person and web-based groups over time. In contrast, there was a significant main effect for time (*F*_2,48_=40; *P*<.001). The pairwise comparisons indicated that, in the in-person group, the knowledge change was reported between the pretest and posttest (contrast=2.14, 95% CI 1.42-2.87; *P*<.001), meaning that knowledge improved immediately following the workshop. In addition, knowledge improved in follow-up evaluations compared with preworkshop knowledge (contrast=1.57, 95% CI 0.84-2.30; *P*<.001). This result revealed an improvement in knowledge regarding the DLW framework at the post- and follow-up evaluations when compared with the baseline scores. In contrast, there was no knowledge change between the posttest and follow-up test (contrast=−0.57, 95% CI −1.30 to 0.16; *P*=.12), which means that knowledge remained the same 3 months after the workshop.

In the web-based group, there was a knowledge change between the pretest and posttest (contrast=2.42, 95% CI 1.70-3.14; *P*<.001), between the pretest and follow-up test (contrast=1.16, 95% CI 0.44-1.88; *P*=.002), and between the posttest and follow-up test (contrast=−1.26, 95% CI −1.97 to −0.54; *P*=.001). Knowledge improved at both the posttest and follow-up evaluations compared with the pretest results. However, the knowledge scores at the follow-up evaluations were lower compared with the posttest results, which means that there was some reduction in knowledge gains over time.

### Quantitative Data: Secondary Outcomes

#### Effects of the Workshops on the Factors Influencing DLW Adoption

Unlike in the knowledge questionnaire, a lower score for the factors influencing DLW adoption did not indicate a wrong answer. Instead, it indicated the degree to which the participants disagreed with the statements in the questionnaire and perceived their capacity to adopt the DLW framework in practice; a higher score meant that the participants were more likely to use the DLW framework in their practice. The mean total score of the pretest for the factors influencing the application of the DLW framework in practice was 38.24 (SD 5.19) out of 60 for the in-person group and 33.82 (SD 6.05) out of 60 for the web-based group. This represented a statistically significant difference using a *t* test between the 2 groups in terms of the factors influencing the application of the DLW framework in practice (*P*=.01). The participants in the in-person group showed higher scores for all questions regarding influencing factors, indicating more positive perceptions of their situations that would support the adoption of the DLW in their practices. Both groups presented the lowest score on the question about how much the participants knew about the DLW framework (in-person=1.95, web-based=1.39), and the highest score was on their willingness to use the DLW framework in practice (in-person=4.9, web-based=4.76). A pretest was conducted before the participants took the DLW workshops, and both groups scored low in terms of their knowledge of the DLW framework, confidence in using it, and how well they knew the resources and experts that would help them understand the DLW framework. The participants felt that the DLW framework would be beneficial in their practice and improve the health outcomes of their clients. They also believed that the DLW framework would fit well in their practice and be easy to apply, and that coworkers would support their use of the DLW framework. The question about how much the participants knew about the DLW resources presented the largest difference in mean scores between the 2 groups. The question about whether the DLW framework would be beneficial in their practice presented the smallest gap between the 2 groups.

Immediately following the completion of the workshop, the mean total score for the factors influencing the use of the new knowledge in practice was 52.10 (SD 4.89) and 43.82 (SD 8.16) out of a maximum score of 60 in the in-person and web-based groups, respectively. Because there was a statistically significant baseline difference in the factors influencing the adoption of the DLW framework between the 2 groups (*P*=.01), the robust regression procedure was conducted using the pretest result as a covariate. The independent variables were the group and the mean total score at pretest, and the dependent variable was the mean total score at posttest. The robust regression result still presented a statistically significant group difference (*F*_2,39_=13.98; *R*^2^=0.5094; *P*=.001) after controlling for the covariate, and the participants in the in-person group presented higher scores on each item of the questionnaire. The in-person group scored an average of 5.17 more points than the web-based group after controlling for the pretest results as a covariate (Table 2).

Compared with the pretest results, both groups had increased scores for every question, except that the participants in the web-based group scored lower on the question regarding how easy it would be to apply the DLW framework in practice. Specifically, both groups presented a large increase in the questions about their knowledge of the DLW framework, confidence in its use, and the extent of their knowledge of its resources and experts compared with the pretest results.

**Table 2:** Robust regression of posttest for factors influencing Do-Live-Well framework adoption.

Variable	*B*^a^ (robust SE; 95% CI)	*t* test (*df*)	*P>|t|*	*F* test (*df*)	*R^2^*
Group	−5.17 (1.48; −8.16 to −2.18)	−3.49 (40)	.001	—^b^	—
Pretest	0.65 (0.14; 0.37 to 0.93)	4.71 (40)	<.001	—	—
Constant	27.09 (5.31; 16.36 to 37.82)	5.11 (40)	<.001	13.98 (2,39)	0.5094

^a^Regression coefficient.

^b^Not available.

The in-person group presented the highest score on the question regarding their willingness to use the DLW framework and the lowest score on the question regarding their confidence in using the DLW framework in their practice. The web-based group presented the highest score on the question regarding the benefit of the DLW framework and the lowest score on the question regarding the ease of using the DLW framework in their practice.

The largest difference between the groups was the question about how well they knew DLW experts; in other words, compared with the web-based group, the participants in the in-person group felt they knew the DLW experts better.

Three months after the workshop, at the follow-up evaluation of the factors influencing the adoption of the DLW framework, the in-person group presented a mean total score of 39.62 (SD 8.24), whereas the web-based group reported a mean total score of 34.77 (SD 8.72) of a maximum score of 60, respectively. The participants in the in-person group scored higher in all items, similar to the pre- and posttest results.

Robust regression was also performed, and no statistically significant difference was noted between the groups after controlling for the covariate (*F*_2,39_=1.69; *R*^2^=0.14; *P*=.19; Table 3). The in-person group presented the highest score on the question regarding their belief in the positive impact of the DLW framework for the health outcomes of their clients and the lowest score on the question about their confidence in using the DLW framework in their practice. The web-based group presented the highest score on the question about their accessibility in the DLW resources and the lowest score on the question about the support of their colleagues in DLW applications.

Both groups presented decreased scores on every question compared with the posttest. The difference in the total mean score of the questions between the 2 groups mostly became smaller compared with the posttest, except for the questions about the benefit of the DLW framework in practice and the support of colleagues in its use. The largest difference between the groups was evident in the question about whether their colleagues would support their DLW application. In other words, the in-person group felt more positive about the support of their colleagues in the DLW application. The smallest difference between the groups was regarding the question about the confidence of the participants in the DLW application; the in-person group’s follow-up scores decreased compared with the posttest results. Throughout all phases (pre-, post-, and follow-up tests), the in-person group presented higher scores for all questions about the factors influencing DLW adoption.

**Table 3:** Robust regression of follow-up results for factors influencing Do-Live-Well framework adoption.

Variable	*B*^a^ (robust SE; 95% CI)	*t* test (*df*)	*P>|t|*	*F* test (*df*)	*R^2^*
Group	−2.73 (2.06; −6.90 to 1.45)	−1.32 (40)	.19	—^b^	—
Pretest	0.44 (0.28; −0.13 to 1.00)	1.56 (40)	.13	—	—
Constant	25.34 (7.85; 9.47 to 41.21)	3.23 (40)	.003	1.69 (2,39)	0.14

^a^Regression coefficient.

^b^Not available.

#### Satisfaction With the Workshops

Immediately following the workshop, the participants in the in-person group were more positive in their appraisal of the workshop (mean total score 106.38, SD 6.73) than the web-based group (mean total score 90.77, SD 16.11). The Mann-Whitney test showed a statistically significant difference between the groups in their satisfaction with the workshop (*P*<.001). The participants in the in-person group scored higher on all items asking about their satisfaction with the workshop. The in-person group was most satisfied with the skills of the instructors in encouraging participant-engagement and least satisfied with the constructive feedback of the instructors.

The web-based group was most satisfied with the accessibility of the learning method and least satisfied with the constructive feedback of the instructors. The largest difference between the groups was regarding the question about the learning environment in favor of the in-person group, and the smallest difference between the groups was with regards to the question about the accessibility of learning.

#### Effects of the Workshops on DLW Application After the Workshops

Three months after the workshop, 43% (9/21) of the people in the in-person group said they had been using the DLW framework. In the web-based group, 27% (6/22) said they had been using the DLW framework. The chi-square test revealed no statistically significant difference in the use of the framework after the workshop (*χ^2^*_1_=1.2; *P*=.28). The clinical practices of the 15 OTs applying DLW concepts from both groups were as follows: mental health (in-person group: 5/6, 83%; web-based group: 1/6, 17%); primary care (in-person group: 2/4, 50%; web-based group: 2/4, 50%); accessibility service (in-person group: 1/1, 100%); pediatrics (web-based group: 1/1, 100%); and private setting (in-person group: 1/1, 100%).

The mean frequency of the DLW framework use with clients was 2.62 (SD 2.54) for the in-person group (n=21) and 1.59 (SD 2.13) for the web-based group (n=22) on a frequency scale of 0-10. The Mann-Whitney test showed no statistically significant difference between the groups (*P*=.13). Regarding the OTs’ frequency of use of the DLW framework other than for their clients (in-person, n=21: mean=2.71/10, SD 2.47; web-based, n=22: mean=1.95/10, SD 2.30), there was no statistically significant difference between the groups (*P*=.22). The results for all outcomes at the 3 time points are presented in Table 4.

**Table 4:** Mean scores for the primary and secondary outcomes at the 3 time points.

Outcomes			Pretest						Posttest							Follow-up test				
			In-person (n=21), mean (SD)		Web-based (n=29), mean (SD)		*P* value		In-person (n=21), mean (SD)		Web-based (n=22), mean (SD)		*P* value		In-person (n=21), mean (SD)			Web-based (n=22), mean (SD)		*P* value
Knowledge regarding DLW^a^			5.48 (1.75)		5.39 (1.69)		.87		7.62 (0.22)		7.81 (0.27)		.57		7.05 (1.12)			6.77 (1.80)		.99
Factors influencing DLW adoption			38.24 (5.19)		33.82 (6.05)		.01		52.10 (4.89)		43.82 (8.16)		.001		39.62 (8.24)			34.77 (8.72)		.19
Reaction to the workshop			N/A^b^		N/A		N/A		106.38 (6.73)		90.77 (16.11)		<.001		N/A			N/A		N/A
**Use**							.17		N/A		N/A		N/A							.28
	Yes	2		0										9			6			
	No	19		29										12			16			
Use with clients (0-10)			N/A		N/A		N/A		N/A		N/A		N/A		2.62 (2.54)			1.59 (2.13)		.13
Use at an organizational level (0-10)			N/A		N/A		N/A		N/A		N/A		N/A		2.71 (2.47)			1.95 (2.30)		.22

^a^DLW: Do-Live-Well.

^b^N/A: not applicable.

### Qualitative Data

#### Participant Characteristics

In total, 18 OTs (9/18, 50% from each group), including 1 man and 17 women, participated in an individual interview an average of 14 weeks after the end of their workshop participation. Their mean age was 39.56 (SD 9.95) years, and their mean work experience was 13.44 (SD 9.57) years. Of the 18 OTs, 4 (22%) had a bachelor’s degree, and 14 (73%) had a master’s degree in Occupational Therapy. From a total of 18 OTs, 10 (56%) applied the DLW framework in their practice, and 8 (44%) did not use it. Their practice settings were as follows: mental health (6/18, 33%), primary care (2/18, 11%), hospital (2/18, 11%), and others (8/18, 44%), including education, long-term care, ophthalmology clinics, pediatric, accessibility, private practices, rehabilitation units, and veterans’ centers.

Five themes from the ideas that were discussed frequently were identified in relation to the OTs’ experience of participating in web-based and in-person workshops, focusing on its facilitators and challenges.

#### Theme 1: Relevance to One’s Practice and Interests May Improve Learning

Participants seemed to engage in learning better when the content was relevant to their practice or interests. In both the web-based and in-person workshops, the learners were able to choose the *case scenario* that was relevant to their practice or interest. Being able to choose the case scenario increased the learners’ motivation. In this regard, one participant in the web-based group said as follows:

I like the fact that I could choose one that was relevant, I think I would have a much harder time obviously with a setting or a population that I am not familiar with. So that was a nice way to learn.

Interviewee 18

In addition, some participants seemed to like *discussions or conversations that were directly related to their practice or interests*. Some found that a downside of the in-person workshop was listening to conversations that were not directly related to their practice or interests. Unlike web-based learning, where people could freely choose what to read based on their interests, people in the in-person workshop had to sit down and listen to every conversation, which could lead to a loss of interest or motivation for learning. One participant in the in-person group said as follows:

I mean, I think sometimes it might have been that people were really passionate about maybe a certain area that I might not have as much interest in, so you would need to certainly wait.

Interviewee 7

#### Theme 2: A Familiar Learning Environment May Facilitate Learning

Some participants felt that they learned better when the learning environment was comfortable. Some participants in the in-person group said that they liked in-person learning because they were *familiar* with its environment. They described in-person learning as *old school* learning where their instructor was physically in front of them. Some said that the in-person workshop was a familiar learning environment, consistent with how they had studied in the past. Thus, for some learners, the familiar learning environment allowed them to easily engage in their learning because that was how they had always learned. Two participants in the in-person group expressed this by saying as follows:

I think it is the familiarity and how I am used to learning because with that I can adapt.

Interviewee 3

Oh, I learn better if the person is actually in front of me.

Interviewee 5

Often with in-person learning, learners are provided with printed materials. During our in-person DLW workshop, we also provided a printed workbook, and this paper-based material seemed to allow learners to better focus on their learning. One participant in the in-person workshop said as follows:

Having paper-based materials typically right in front of me as well is helpful. That is how I typically retain information better. This brain of mine functions better.

Interviewee 9

An electronic version of the workbook was provided to participants in the web-based workshop. One participant in the web-based workshop felt less familiar with the web-based learning environment and used her own learning strategy to overcome the challenges she experienced. The participant mentioned that it was not easy for her to go back and forth between the webpages to find an appropriate reference to answer the discussion questions. Thus, she used her own notes and wrote down the key point of the lecture, which she used to answer the discussion questions. In this way, she made the web-based context more familiar to her own learning style to enhance her engagement with the material. She said the following:

I do like the website format and kind of like typing out responses, but a downside to that is that I kind of always had to reference material from different pages to look at my answers again. What I found helpful is just like I just kind of write my own notes on the side and I refer to that when I write the answers.

Interviewee 13

#### Theme 3: Synchronous Interaction Is Valuable in the Learning Process

Participants in both the web-based and in-person workshops found synchronous interaction to be a great facilitator of their learning. They mentioned that *nonverbal communication cues* were important in their learning. One participant said as follows:

I feel like the in-person, the face-to-face interactions would allow me to take in cues that you may not necessarily be able to get when you are doing even the phone call or teleconference. I truly believe that there is a lot of information in nonverbal communication.

Interviewee 8

In addition, *dynamic discussions* seemed to be another important aspect of learning, whereby learners actively exchanged opinions with peers and instructors on various topics regarding the DLW framework. This active process of sharing thoughts exposed them to different perspectives that they had not previously encountered. One participant shared her thoughts regarding dynamic discussions:

I think that for me it is the discussions, from hearing others’ point of view, and then how other people apply it to situations that I might not even have thought of.

Interviewee 3

In contrast*,* one participant in the web-based group said that there was no opportunity for dynamic discussions in web-based learning:

[In online learning] you cannot build as much on top of other people’s things. So, you get to see more of what people are saying, but you cannot brainstorm together.

Interviewee 14

Furthermore, being able to *ask questions* the moment they had them was another facilitator in the participants’ learning. If learners had questions about the content, the learners in the in-person group could immediately ask the instructor. However, unlike the in-person learning environment, it was not easy to ask a question in real time through the web-based learning platform. One participant in the web-based group said as follows:

Because it [online learning] was offered asynchronously you did not necessarily have a chance to ask a question at the moment if there was a question.

Interviewee 15

Similarly, participants liked to receive *immediate feedback* from peers or instructors during their learning. One participant in the in-person group said:

I really liked to have immediate feedback from not just the peers but also the organizers of the workshop.

Interviewee 8

Finally, the learners in the in-person workshop liked to *meet other OTs* from different practice settings. One participant in the in-person group said as follows:

I really enjoyed meeting other people in that course and seeing what they are doing in their practice. I think a lot of them had a unique OT role and also, how they are using the Do-Live-Well method.

Interviewee 5

In contrast, one participant in the web-based group expressed that the web-based workshop did not provide the same quality networking opportunities as the in-person workshop:

The disadvantage [of online learning] is that you do not necessarily get that face-to-face networking quality.

Interviewee 18

#### Theme 4: Ease of Access to Learning Should Be Considered

Accessibility to learning seemed to be an important aspect that educators should consider when providing educational opportunities. The participants in both the web-based and in-person workshop groups identified some benefits and challenges of accessing each learning format.

First, the participants in the in-person workshop group mentioned that *commuting* was a challenge in accessing the workshop location. For learners who did not have cars, commuting to the workshop location was difficult. In addition, the cold winter weather in Canada affected their access to learning. Two participants in the in-person group commented the following:

The challenge is the commute time. Driving there, at the parking, getting the day off work to do it.

Interviewee 1

I think the weather was not that nice. It was cold. I mean the commute was not that bad from Toronto to Hamilton but obviously, that would have deterred quite a few people if they do not have a car or it is too far to be able to access.

Interviewee 5

Some participants in the web-based workshop group mentioned that the web-based workshop was a *safe way* of learning. Owing to the COVID-19 pandemic, web-based education has been considered a safe and primary route by which learners can take courses without worrying about risks. One participant in the web-based group said:

I think benefits of online is that, like especially in this COVID season, you can be safe and like kind of not be at risk of being exposed to COVID for sure.

Interviewee 13

In addition, learners in the web-based group said that a benefit of web-based learning was that it was *free from geographical restrictions*. Some learners took the web-based courses in Alberta and even while traveling outside of Canada; thus, learners took courses wherever they had internet access, which made learning more accessible for them. One participant in the web-based group expressed as follows:

I am in Kingston...being able to take it here and in Argentina, that was beneficial.

Interviewee 14

However, if the learner did not have the necessary *equipment* to take the web-based class, such as internet access and a computer, there were restrictions on taking the course itself, which affected learning. Regarding this equipment requirement and its inherent challenges, a participant in the web-based group said: “It was finding a computer that I can use because I do not have my own computer” [Interviewee 10].

#### Theme 5: Flexibility in Web-Based Learning Can Be Both Beneficial and Challenging

According to the opinions of the participants in the web-based workshop group, the flexibility of web-based learning seemed to be both an advantage and a disadvantage. First, *self-paced* learning was found to be a facilitator of their learning process. In web-based learning, learners could choose the best time of the day to take the course, which possibly decreased potential distractions. Moreover, learners were able to control the speed of learning based on their individual learning styles. A participant in the web-based group shared her thoughts:

I would say that you can do it at your own pace. So if you have a setting like I do, where you can have interruptions, you think you might have a certain amount of time to set aside, but you then are interrupted with something that you would like to do or it needs to be done, that you can go ahead and do that, and then you can continue your learning.

Interviewee 10

Another benefit of web-based learning was *repeatability*. In web-based learning, learners could repeat the course whenever they wanted. For example, they could repeat the specific content that they did not understand well, and this ability to repeat the course helped learners better understand and remember the content. One participant in the web-based group shared her experience of being able to repeat the content:

I liked that I could actually review the videos. I went back to watch them a few times to remind myself what you think. I think I actually went back with one of the later parts of it and went back and watched it again one of the earlier ones. I like that aspect to which I do not think you could do in an in-person setting. You would have to just remember what was happening.

Interviewee 16

However, the flexibility of learning also hindered the learning process because some learners *procrastinated* on completing the course. The learners postponed taking the web-based course for various reasons. One participant in the web-based group said:

I think I procrastinate. I think it is easier to not set a time to do it. Whereas if it is in-person you are just there. You do not have an option. Okay, you go. For the most part or that is the only time they are offering it. So that is the time you have to get up.

Interviewee 14

Some participants also had difficulty *prioritizing* taking the web-based course over other tasks, which affected their overall engagement in learning. A participant in the web-based group expressed the difficulty of prioritizing as follows:

So, for me, making it a priority was a bit of a challenge, because I had the flexibility to do it whenever, I did end up doing most of it like the night before it closed. So that was not necessarily how I had anticipated being able to use it. Because of that, my participation in the online forums was pretty minimal.

Interviewee 12

## Discussion

### Principal Findings

Considering the appeal and current popularity of web-based learning, we examined the effectiveness of a web-based PBL-based DLW workshop compared with a PBL-based in-person DLW workshop. We also gained insights into learners’ perspectives on their participation in both learning formats. The quantitative data showed no statistically significant difference between the groups in knowledge change at the 3 time points (pre-, post-, and follow-up testing), but there was a reduction in knowledge over time in the web-based group. A statistically significant difference was present in factors influencing DLW adoption and satisfaction with the workshop at posttest. However, there was also no difference in the use of the DLW framework 3 months after the workshops. We also identified the key aspects of the learning experience of the participants through our qualitative data: relevance to practice and interest, a familiar learning environment, synchronous in-person interaction, ease of access to learning, and flexibility in web-based learning.

Similar to a recent review of the effectiveness of web-based learning compared with traditional in-person learning for health care professionals [17], the quantitative results about knowledge change showed no differences in knowledge gained between the groups [17]. This suggests that web-based learning is as promising as traditional learning for obtaining knowledge. Undoubtedly, acquiring knowledge is important for health care professionals, as they need foundational knowledge to solve various clinical problems in practice [31]. The participants in our study who attended the in-person workshop had a more satisfying learning experience in all aspects of the workshop based on our quantitative results. Bray et al [32] identified that learners considered interaction as an important factor that led to learning satisfaction. This is reinforced by our qualitative findings, in which participants highlighted the importance of interaction with instructors and peers in the learning process. There were no synchronous interactions in the web-based workshop in our study; thus, as shown by our satisfaction results, the participants in the web-based groups who felt the lack of personal interactions might have been less satisfied with the workshop. In addition, this aspect of social interaction may influence the long-term effect of knowledge retention. This study reported a reduction in knowledge in the web-based group over time, albeit not statistically significant. Real-time social interactions have reported the effectiveness of learning by helping learners “organize their thoughts, reflect on their understanding, and find gaps in their reasoning” [33]. Thus, a lack of synchronous interactions with peers and instructors may negatively impact the knowledge retention and satisfaction of the learners in the web-based group.

Regarding the factors influencing the DLW concepts in practice, immediately after the workshop, the participants in the in-person workshop seemed to be more positive toward the DLW application in their practice; however, 3 months after the workshop, there was no statistically significant difference in the factors influencing DLW adoption between the groups. At the time of the research, the COVID-19 pandemic resulted in significant disruptions in the practice contexts of the OTs, and learners’ perceptions of the DLW application might have been affected by the COVID-19 pandemic. The participants who believed that the DLW could be incorporated into their practice faced barriers to its use during COVID-19 pandemic restrictions and changes to their practice. Many in-person programs were canceled, and OTs were busy dealing with urgent situations and changed policies, which may have resulted in decisions not to implement DLW concepts as planned.

Immediately after the DLW workshops, there was the largest difference between the 2 groups regarding the question about how well the participants knew the DLW experts. Compared with the in-person workshop, where the participants could meet and talk with the DLW experts, the participants in the web-based group may have given this question a lower score because they did not have the same opportunity to meet the experts in person. However, this difference between the 2 groups did not last 3 months after the workshops, as indicated by the decreased score in the in-person group. Only 1 person from the web-based group contacted the DLW team after the workshop, and it is expected that even though the participants in the in-person group believed they knew the DLW experts well immediately after the workshop, this impression did not last for 3 months because they did not maintain connections with the experts after the workshop. A recent survey study of the preferences of OTs in continuing education shows that OTs want to receive ongoing individual support even after their education has ended [34]. Thus, we recommend that educators provide a way for learners to stay connected with experts in new knowledge even after disseminating the knowledge. A possible way to connect learners and experts is mentorship. Mentor-mentee programs have been used in occupational therapy education to support the growth of less experienced OTs in professional skills [35,36]. A case study reported that a novice OT found mentorship helpful in applying knowledge to real-world practice, leading to the professional growth of the OT [36]. Thus, having a regular meeting or follow-up check-in opportunity may allow learners to feel connected to the DLW experts, enabling them to sustain their knowledge and support them in applying what they have learned.

The relevance of knowledge to clinical practice and interest was emphasized in our qualitative findings. Regardless of the type of workshop learners participated in, quantitative and qualitative findings suggest that being able to choose a case scenario related to their practice and interest was helpful in their learning process. In a review of learning theories and education for health care professionals, Abela argues that the relevance of new knowledge to learners’ clinical practice should be considered when educators decide on discussion topics [37]. Furthermore, Gewurtz et al [38] also noted that PBL is premised on the assumption that “learning is most effective when it is applicable to practice” [38]. Therefore, educators planning to develop web-based and in-person learning for OTs should reflect on how new knowledge is relevant to the learners’ practice.

Our quantitative results revealed that in-person learners appreciated the various elements of the satisfaction questionnaire more positively. This may be the result of the learning preferences of the participants before attending the workshops; both the in-person and web-based groups preferred in-person learning at the pretest. Web-based learners who preferred the in-person learning format may have been less satisfied with the web-based learning format.

In the satisfaction questionnaire, the accessibility of web-based learning was the component with which web-based learners were most satisfied. In the literature, accessibility has been recognized as a great benefit of web-based learning by allowing anyone to access learning materials without restrictions [39]. This benefit of accessibility was made more evident by our qualitative findings. The web-based workshop participants appreciated that they could participate in learning without regional restrictions. Even when traveling abroad during the study period, a participant could take the web-based DLW courses. The benefit of this accessibility would make learning easier for international learners or learners in remote areas who want to learn more about the DLW framework. Therefore, web-based education will help educational institutions or associations that want to attract global learners. Access to reliable internet and web-based learning equipment is important for web-based learning [40]. Since the COVID-19 outbreak, many people have been working from home or taking web-based courses. If a person does not have their own computer and instead shares one with other family members, they may need to wait until the other family members finish using the computer, which may prevent a person from accessing the web-based courses. Thus, access to internet and web-based learning equipment should be considered for web-based learners.

The learners in the web-based group valued the flexibility provided by web-based learning, given that they could take and repeat the modules whenever they wanted because the workshop materials were provided asynchronously. The benefits of the asynchronous feature of web-based learning were that it supported different learning styles and preferences [41]. However, web-based learners stated that the flexibility of web-based learning also hindered their learning. Participants in the web-based workshop found it difficult to prioritize web-based learning over other tasks. Adult learners have responsibilities at home and at work, and they are often placed in a variety of situations that impede learning [42]. Thus, the flexibility of web-based learning seemed to allow learners to prioritize other tasks over web-based courses, resulting in them not having enough time to take the courses. In both the post-and follow-up evaluations, 7 people did not complete the evaluations. Although it was not known whether the participants who did not complete the evaluations completed the web-based courses, the dropout rate in the web-based group may indicate that the flexibility of the web-based learning environment could negatively affect the completion of web-based courses. Moreover, web-based learners in this study seemed to procrastinate in the web-based course; learners’ procrastination has been a major disadvantage of web-based learning [43] and it has a negative effect on learners’ perceptions of the effectiveness of web-based learning [44].

In our qualitative findings, the lack of ease in networking with others was identified as a challenge of web-based learning. New knowledge is disseminated through communication channels within a social system [26], and educators would need to think of providing the best way to enable learners to communicate with educators and their peers. In our study, although we provided an internet-based space for web-based learners to communicate with each other, the quality of asynchronous communication may be different from that of synchronous communication. The importance of synchronous interactions was emphasized through the interviews with participants in both the web-based and in-person workshops. Thus, adding synchronous communication to web-based learning may benefit learners by encouraging them to engage in their learning more actively. In the literature, an opportunity to have synchronous communication allowed learners to discuss the content in-depth and kept them feeling an urgency for learning [45] and, therefore, may contribute to the successful completion of web-based courses. Furthermore, synchronous communication is more related to the social aspect of learning than asynchronous communication [46]. Considering that OTs value the social aspect of learning [16], future research on continuing education for OTs should include synchronous discussions via video conferences or live chats to maximize benefits. By doing so, learners may have more time to absorb and reflect on what they have learned and to enhance and validate their understanding by asking questions and receiving immediate feedback.

### Strengths

To our knowledge, no studies have examined the effectiveness of web-based continuing learning with a comparison group of in-person learners specifically for OTs. This study provided quantitative findings, and the authors were able to directly hear the perspectives and learning experiences of the participants in both web-based and in-person learning environments. We believe this study can support occupational therapy educators in developing and providing effective web-based education by understanding the advantages and disadvantages of the 2 different educational methods.

### Limitations

The web-based workshop platform allowed us to identify which participants joined the discussion forums and to see their login information via the workshop website, but we did not know if the participants completed all the course materials. Although we assumed that those who did not complete the postevaluation might not have completed the web-based course, postworkshop evaluation is not an accurate indicator of successful completion of the course. Thus, for future educational studies examining the effectiveness of web-based education, researchers should track learners’ course completion, if possible. Unless preinstalled software to track learners’ completion is available, researchers may need to ask the participants directly about course completion. In addition, all questionnaires used to measure the outcomes of this study were developed specifically for this study, and thus the reliability and validity of the questionnaires themselves have not been demonstrated. Future studies could focus on developing standard measures to evaluate the effectiveness of educational interventions. In addition, this study was conducted in Hamilton, Canada, but participants were recruited from across Canada. We were not able to randomize the participants because OTs far from the study site could not be included in the in-person group. Future studies may consider offering both web-based and in-person workshops to all participants and then randomize them.

### Conclusions

This study suggests that web-based education can be effective for OTs, as web-based education enables learners to acquire a similar level of knowledge compared with in-person education. In addition, each educational method has strengths and barriers identified by the learners. Adding a synchronous feature and a mentor or individual follow-up to web-based learning may facilitate more active involvement by participants in their learning, resulting in a more positive web-based learning experience.

## Appendix

Multimedia Appendix 1 Prequestionnaire.

Multimedia Appendix 2 Postquestionnaire.

Multimedia Appendix 3 Follow-up questionnaire.

## Abbreviations

DLW: Do-Live-Well
OT: occupational therapist
PBL: problem-based learning
