# Supplementary material for: Comparing Web-Based and In-Person Educational Workshops for Canadian Occupational Therapists and Understanding Their Learning Experiences: Mixed Methods Study
Source: JMIR Med Educ. 2022 Jan 4;8(1):e31634. doi: 10.2196/31634 (PMC8767476; doi:10.2196/31634)
Supplement: Multimedia Appendix 2 [file mededu_v8i1e31634_app2.docx]

**Post Questionnaire**

**Part 1. Factors Influencing Adoption of the DLW framework and Intention to Use it**

**Please rate the extent to which you agree with the following statements.**

1. I know a lot about the DLW framework.

| Strongly Disagree | Disagree | Slightly  Disagree | Slightly  Agree | Agree | Strongly  Agree |
| --- | --- | --- | --- | --- | --- |
| (1) | (2) | (3) | (4) | (5) | (6) |
|  |  |  |  |  |  |

2. Applying the DLW framework will be beneficial for me as a clinician.

| Strongly Disagree | Disagree | Slightly  Disagree | Slightly  Agree | Agree | Strongly  Agree |
| --- | --- | --- | --- | --- | --- |
| (1) | (2) | (3) | (4) | (5) | (6) |
|  |  |  |  |  |  |

3. The DLW framework will fit well into my clinical setting.

| Strongly Disagree | Disagree | Slightly  Disagree | Slightly  Agree | Agree | Strongly  Agree |
| --- | --- | --- | --- | --- | --- |
| (1) | (2) | (3) | (4) | (5) | (6) |
|  |  |  |  |  |  |

4. The DLW framework will be easy for me to apply in my practice.

| Strongly Disagree | Disagree | Slightly  Disagree | Slightly  Agree | Agree | Strongly  Agree |
| --- | --- | --- | --- | --- | --- |
| (1) | (2) | (3) | (4) | (5) | (6) |
|  |  |  |  |  |  |

5. I feel confident in applying the DLW framework in my practice.

| Strongly Disagree | Disagree | Slightly  Disagree | Slightly  Agree | Agree | Strongly  Agree |
| --- | --- | --- | --- | --- | --- |
| (1) | (2) | (3) | (4) | (5) | (6) |
|  |  |  |  |  |  |

6. Applying the DLW framework in my practice will improve clients’ health and well-being.

| Strongly Disagree | Disagree | Slightly  Disagree | Slightly  Agree | Agree | Strongly  Agree |
| --- | --- | --- | --- | --- | --- |
| (1) | (2) | (3) | (4) | (5) | (6) |
|  |  |  |  |  |  |

7. My colleagues will support me to use the DLW framework in my practice.

| Strongly Disagree | Disagree | Slightly  Disagree | Slightly  Agree | Agree | Strongly  Agree |
| --- | --- | --- | --- | --- | --- |
| (1) | (2) | (3) | (4) | (5) | (6) |
|  |  |  |  |  |  |

8. I know of resources that can help me better understand about the DLW framework.

| Strongly Disagree | Disagree | Slightly  Disagree | Slightly  Agree | Agree | Strongly  Agree |
| --- | --- | --- | --- | --- | --- |
| (1) | (2) | (3) | (4) | (5) | (6) |
|  |  |  |  |  |  |

9. I know experts in the DLW framework.

| Strongly Disagree | Disagree | Slightly  Disagree | Slightly  Agree | Agree | Strongly  Agree |
| --- | --- | --- | --- | --- | --- |
| (1) | (2) | (3) | (4) | (5) | (6) |
|  |  |  |  |  |  |

10. I would like to use the DLW framework in my practice.

| Strongly Disagree | Disagree | Slightly  Disagree | Slightly  Agree | Agree | Strongly  Agree |
| --- | --- | --- | --- | --- | --- |
| (1) | (2) | (3) | (4) | (5) | (6) |
|  |  |  |  |  |  |

11. Please provide any additional relevant information in the box below.

|  |
| --- |

**Part 2: Knowledge Questions**

- Multiple Choice Questions (choose the correct answer)

1. Which of the following is *not* one of the DLW dimensions of experience?

(1) Activating your body, mind, and senses

(2) Contributing to community and society

(3) Taking care of yourself

(4) Saving your energy

(5) Building prosperity

2. Which of the following is *not* one of the DLW activity patterns?

(1) Routine

(2) Control/choice

(3) Engagement

(4) Meaning

(5) Collaboration

- True or False Questions (choose the correct answer)

1. There are three main sections in the DLW framework: dimensions of experience, activity patterns, and health and well-being outcomes. (T / F)

2. Activity patterns consider the nature of what people do but do not necessarily consider how people engage in day-to-day activities. (T / F)

3. Although eight dimensions of experience are intended to be discrete, they are interrelated. (T / F)

4. The DLW framework is designed to be prescriptive so that clinicians can easily and accurately apply its concepts in their practice. (T / F)

5. Patterns of activity engagement affect the extent to which positive health and well-being outcomes are met. (T / F)

6. According to the DLW framework, there are two health and wellness outcomes; physical and mental health. (T / F)

7. The DLW framework is designed to promote reflection and occupational engagement by acknowledging the outcomes of day-to-day activities are always positive. (T / F)

8. The DLW framework is a conceptual model and can be applied at the three levels: an individual, community, and national level. (T / F)

**Part 3: Reactions to the workshops**

Instruction

Please respond to the following statements by using the 7-point rating scale to indicate the extent to which you agree or disagree with each statement. Please click the number that applies.

7= Strongly agree / 6 = agree / 5= Slightly agree / 4= Neutral / 3=Slightly disagree / 2= disagree / 1=Strongly disagree

1. The accessibility of the workshop was convenient.

| Strongly disagree | 1 | 2 | 3 | 4 | 5 | 6 | 7 | Strongly agree |
| --- | --- | --- | --- | --- | --- | --- | --- | --- |

2. The learning environment encouraged me to actively participate in learning.

| Strongly disagree | 1 | 2 | 3 | 4 | 5 | 6 | 7 | Strongly agree |
| --- | --- | --- | --- | --- | --- | --- | --- | --- |

3. The time frame of the workshop was appropriate.

| Strongly disagree | 1 | 2 | 3 | 4 | 5 | 6 | 7 | Strongly agree |
| --- | --- | --- | --- | --- | --- | --- | --- | --- |

4. The content was helpful to understand the DLW framework.

| Strongly disagree | 1 | 2 | 3 | 4 | 5 | 6 | 7 | Strongly agree |
| --- | --- | --- | --- | --- | --- | --- | --- | --- |

5. The case studies were helpful.

| Strongly disagree | 1 | 2 | 3 | 4 | 5 | 6 | 7 | Strongly agree |
| --- | --- | --- | --- | --- | --- | --- | --- | --- |

6. The level of the workshop was appropriate.

| Strongly disagree | 1 | 2 | 3 | 4 | 5 | 6 | 7 | Strongly agree |
| --- | --- | --- | --- | --- | --- | --- | --- | --- |

7. My learning objectives were achieved.

| Strongly disagree | 1 | 2 | 3 | 4 | 5 | 6 | 7 | Strongly agree |
| --- | --- | --- | --- | --- | --- | --- | --- | --- |

8. The workshop met my expectations.

| Strongly disagree | 1 | 2 | 3 | 4 | 5 | 6 | 7 | Strongly agree |
| --- | --- | --- | --- | --- | --- | --- | --- | --- |

9. The learning resources were appropriate.

| Strongly disagree | 1 | 2 | 3 | 4 | 5 | 6 | 7 | Strongly agree |
| --- | --- | --- | --- | --- | --- | --- | --- | --- |

10. The learning resources were helpful.

| Strongly disagree | 1 | 2 | 3 | 4 | 5 | 6 | 7 | Strongly agree |
| --- | --- | --- | --- | --- | --- | --- | --- | --- |

11. The amounts of learning resources were sufficient.

| Strongly disagree | 1 | 2 | 3 | 4 | 5 | 6 | 7 | Strongly agree |
| --- | --- | --- | --- | --- | --- | --- | --- | --- |

12. The instructor had a good understanding of the topics.

| Strongly disagree | 1 | 2 | 3 | 4 | 5 | 6 | 7 | Strongly agree |
| --- | --- | --- | --- | --- | --- | --- | --- | --- |

13. The instructor had a good skill to encourage participant engagement.

| Strongly disagree | 1 | 2 | 3 | 4 | 5 | 6 | 7 | Strongly agree |
| --- | --- | --- | --- | --- | --- | --- | --- | --- |

14. The instructor provided sufficient feedback.

| Strongly disagree | 1 | 2 | 3 | 4 | 5 | 6 | 7 | Strongly agree |
| --- | --- | --- | --- | --- | --- | --- | --- | --- |

15. The instructor provided constructive feedback.

| Strongly disagree | 1 | 2 | 3 | 4 | 5 | 6 | 7 | Strongly agree |
| --- | --- | --- | --- | --- | --- | --- | --- | --- |

16. I recommend that the workshop be repeated for other occupational therapists.

| Strongly disagree | 1 | 2 | 3 | 4 | 5 | 6 | 7 | Strongly agree |
| --- | --- | --- | --- | --- | --- | --- | --- | --- |

Please provide any suggestions for improvement, comments, and feedback on this workshop in the box below.

|  |
| --- |

What additional content would you like us to cover in the workshop?

|  |
| --- |

For participants of online workshop, please let us know if there were any technical issues you experienced while taking this online workshop.

|  |
| --- |
