# Supplementary material for: Comparing Web-Based and In-Person Educational Workshops for Canadian Occupational Therapists and Understanding Their Learning Experiences: Mixed Methods Study
Source: JMIR Med Educ. 2022 Jan 4;8(1):e31634. doi: 10.2196/31634 (PMC8767476; doi:10.2196/31634)
Supplement: Multimedia Appendix 3 [file mededu_v8i1e31634_app3.docx]

**Follow-up Questionnaire**

**Part 1. Current Status of the Use of the DLW framework**

Are you currently using the DLW framework? **Y** or **N**

If **yes**,

1. On a scale from 0-10, how often do you use the DLW framework with your clients?

| 0 | 1 | 2 | 3 | 4 | 5 | 6 | 7 | 8 | 9 | 10 |
| --- | --- | --- | --- | --- | --- | --- | --- | --- | --- | --- |

0: I never use the DLW framework with my clients

5: I use the DLW framework with the about half of my clients

10: I use the DLW framework with all of my clients

2. On a scale from 0-10, how often do you apply the DLW framework in your practice? (This includes things of varying scope you do in your practice except for the direct use with your clients. E.g., documentation, team communication, collaborative work with other disciplines, or writing a referral form)

| 0 | 1 | 2 | 3 | 4 | 5 | 6 | 7 | 8 | 9 | 10 |
| --- | --- | --- | --- | --- | --- | --- | --- | --- | --- | --- |

0: I never use the DLW framework in my practice

5: I use the DLW framework in my practice at about 50% rate

10: I use the DLW framework in my practice all the time

**Part 2. Factors Influencing Adoption of the DLW framework and Use of it**

**Please rate the extent to which you agree with the following statements.**

1. I know a lot about the DLW framework.

| Strongly Disagree | Disagree | Slightly  Disagree | Slightly  Agree | Agree | Strongly  Agree |
| --- | --- | --- | --- | --- | --- |
| (1) | (2) | (3) | (4) | (5) | (6) |
|  |  |  |  |  |  |

2. Applying the DLW framework is beneficial for me as a clinician.

| Strongly Disagree | Disagree | Slightly  Disagree | Slightly  Agree | Agree | Strongly  Agree |
| --- | --- | --- | --- | --- | --- |
| (1) | (2) | (3) | (4) | (5) | (6) |
|  |  |  |  |  |  |

3. The DLW framework fits well into my clinical setting.

| Strongly Disagree | Disagree | Slightly  Disagree | Slightly  Agree | Agree | Strongly  Agree |
| --- | --- | --- | --- | --- | --- |
| (1) | (2) | (3) | (4) | (5) | (6) |
|  |  |  |  |  |  |

4. The DLW framework is easy for me to apply in my practice.

| Strongly Disagree | Disagree | Slightly  Disagree | Slightly  Agree | Agree | Strongly  Agree |
| --- | --- | --- | --- | --- | --- |
| (1) | (2) | (3) | (4) | (5) | (6) |
|  |  |  |  |  |  |

5. I feel confident in applying the DLW framework in my practice.

| Strongly Disagree | Disagree | Slightly  Disagree | Slightly  Agree | Agree | Strongly  Agree |
| --- | --- | --- | --- | --- | --- |
| (1) | (2) | (3) | (4) | (5) | (6) |
|  |  |  |  |  |  |

6. Applying the DLW framework in my practice improves clients’ health and well-being.

| Strongly Disagree | Disagree | Slightly  Disagree | Slightly  Agree | Agree | Strongly  Agree |
| --- | --- | --- | --- | --- | --- |
| (1) | (2) | (3) | (4) | (5) | (6) |
|  |  |  |  |  |  |

7. My colleagues support me to use the DLW framework in my practice.

| Strongly Disagree | Disagree | Slightly  Disagree | Slightly  Agree | Agree | Strongly  Agree |
| --- | --- | --- | --- | --- | --- |
| (1) | (2) | (3) | (4) | (5) | (6) |
|  |  |  |  |  |  |

8. I know of resources that can help me better understand about the DLW framework.

| Strongly Disagree | Disagree | Slightly  Disagree | Slightly  Agree | Agree | Strongly  Agree |
| --- | --- | --- | --- | --- | --- |
| (1) | (2) | (3) | (4) | (5) | (6) |
|  |  |  |  |  |  |

9. I know experts in the DLW framework.

| Strongly Disagree | Disagree | Slightly  Disagree | Slightly  Agree | Agree | Strongly  Agree |
| --- | --- | --- | --- | --- | --- |
| (1) | (2) | (3) | (4) | (5) | (6) |
|  |  |  |  |  |  |

10. Please provide any additional relevant information in the box below.

|  |
| --- |

**Part 3: Knowledge Questions**

- Multiple Choice Questions (choose the correct answer)

1. Which of the following is *not* one of the DLW dimensions of experience?

(1) Activating your body, mind, and senses

(2) Contributing to community and society

(3) Taking care of yourself

(4) Saving your energy

(5) Building prosperity

2. Which of the following is *not* one of the DLW activity patterns?

(1) Routine

(2) Control/choice

(3) Engagement

(4) Meaning

(5) Collaboration

- True or False Questions (choose the correct answer)

1. There are three main sections in the DLW framework: dimensions of experience, activity patterns, and health and well-being outcomes. (T / F)

2. Activity patterns consider the nature of what people do but do not necessarily consider how people engage in day-to-day activities. (T / F)

3. Although eight dimensions of experience are intended to be discrete, they are interrelated. (T / F)

4. The DLW framework is designed to be prescriptive so that clinicians can easily and accurately apply its concepts in their practice. (T / F)

5. Patterns of activity engagement affect the extent to which positive health and well-being outcomes are met. (T / F)

6. According to the DLW framework, there are two health and wellness outcomes; physical and mental health. (T / F)

7. The DLW framework is designed to promote reflection and occupational engagement by acknowledging the outcomes of day-to-day activities are always positive. (T / F)

8. The DLW framework is a conceptual model and can be applied at the three levels: an individual, community, and national level. (T / F)
